# Microtubule Severing Protein Fignl2 Contributes to Endothelial and Neuronal Branching in Zebrafish Development

**DOI:** 10.3389/fcell.2020.593234

**Published:** 2021-01-18

**Authors:** Zhangji Dong, Xu Chen, Yuanyuan Li, Run Zhuo, Xiaona Lai, Mei Liu

**Affiliations:** Key Laboratory of Neuroregeneration of Jiangsu and Ministry of Education, Co-innovation Center of Neuroregeneration, Nantong University, Nantong, China

**Keywords:** development, branching, neuron, vascular endothelial cells, fidgetin-like 2

## Abstract

Previously, *fidgetin* (*fign*) and its family members *fidgetin-like 1* (*fignl1*) and *fidgetin-like 2* (*fignl2*) were found to be highly expressed during zebrafish brain development, suggesting their functions in the nervous system. In this study, we report the effects of loss-of-function of these genes on development. We designed and identified single-guide RNAs targeted to generate *fign, fignl1*, and *fignl2* mutants and then observed the overall morphological and behavioral changes. Our findings showed that while *fign* and *fignl1* null mutants displayed no significant defects, *fignl2* null zebrafish mutants displayed pericardial edema, reduced heart rate, and smaller eyes; *fignl2* null mutants responded to the light-darkness shift with a lower swimming velocity. *fignl2* mRNAs were identified in vascular endothelial cells by *in situ* hybridization and re-analysis of an online dataset of single-cell RNAseq results. Finally, we used morpholino oligonucleotides to confirm that *fignl2* knockdown resulted in severe heart edema, which was caused by abnormal vascular branching. The zebrafish *fignl2* morphants also showed longer axonal length and more branches of caudal primary neurons. Taken together, we summarize that Fignl2 functions on cellular branches in endothelial cells and neurons. This study reported for the first time that the microtubule-severing protein Fignl2 contributes to cell branching during development.

## Introduction

Morphology and motility of a cell are determined by the regulation of the cytoskeleton, especially microtubules. Microtubules form highly complex and dynamic arrays that play roles in various aspects of the development and function of cells. Microtubule dynamics are regulated by microtubule-associated proteins, of which microtubule severing proteins (MSPs) such as spastin, katanin, and fidgetin are key regulatory factors of microtubule dynamics (Karabay et al., 2004; Butler et al., 2010; Leo et al., 2015; Menon and Gupton, 2016). These MSPs are members of the AAA (ATPase family associated with various cellular activities) family, which is capable of severing microtubules into short fragments by forming a hexamer that consumes ATP (Sharp and Ross, 2012; McNally and Roll-Mecak, 2018).

Studies on katanin and spastin demonstrated that they primarily cut stable long microtubules into short ones for microtubule transport or elongation at new generating plus ends, while fidgetin (Fign) and its family members may have more functions. *fign* mutation resulted in mice “fidget” behavior (Yang et al., 2005); human FIGN suppressed microtubule growth via minus-end depolymerization during cell division (Mukherjee et al., 2012); FIGNL1 is regarded to regulate DNA homologous recombination repair (Yuan and Chen, 2013; Kumar et al., 2019) or meiotic crossovers (Girard et al., 2015); Charafeddine et al. reported that Fign12 modulates orientation of cell migration by shearing microtubules (Charafeddine et al., 2015), and O'Rourke el al. showed that Fign12 affected wound healing (O'Rourke et al., 2019). We previously studied Fign's function in rat brain astrocytes and found that Fign depletion resulted in a remarkable increase in tyrosine-modified microtubules, and thus changed the microtubule orientation in the cell cortical region (Hu et al., 2017). Leo et al. reported that Fign knockout in mice neurons increased the unacetylated microtubule mass (Leo et al., 2015). However, Drosophila Fign facilitates microtubule disassembly in dendrites but not in axons after neuron injury (Tao et al., 2016). These inconsistent functional features of Fign family members in different species or cell types have aroused our interest. Therefore, we attempted to comparatively study the functions of Fign family members in one animal model.

Similar to mice, rats and humans, there are three Fign paralogues, namely Fign, Fidgetin-like 1 (Fignl1), and Fidgetin-like 2 (Fignl2) in zebrafish. In the literature on Fign family members' functions, only Fignl1 was reported to be enriched in axons and growth cones of neurons and to play roles in the motor circuit in zebrafish larvae (Fassier et al., 2018), and Fignl1 overexpressed in zebrafish embryos inhibited ciliogenesis and decreased ciliary length (Zhao et al., 2016); however, the functions of Fign and Fignl2 remain elusive. Recently, we investigated the expression patterns of *fign* and its family members, *fidgetin-like 1* (*fignl1*) and *fidgetin-like 2* (*fignl2*), during zebrafish embryonic development, and *fign* genes were found highly expressed during brain development, suggesting their functions in the nervous system (Dong et al., 2021). In this study, we aim to compare the developmental features after loss-of-function of these genes to further clarify their functions and possible mechanism.

## Results

### Generation and Phenotype Analysis of Mutants of *fign, fignl1*, and *fignl2*

CRISPR/Cas9 was used to generate mutants harboring new alleles of Fign, Fignl1, and Fignl2 that contain frame-shift mutations. Selected target sites are shown in Table 1 as sense strand genomic sequences. Incross was performed in the F1 generation to produce homozygous mutant F2 and siblings of the other two genotypes shown in this study.

**Table 1:** sgRNAs used in this study.

**sgRNA**	**Gene**	**Position in gene**	**Sequence**	**Location in CDS**	**Mutation^**#**^**
Fign-sgRNA1	*Fign* [ENSDARG00000008662]	150565–150584	CTTACAGCGGCGGTCAAAGC		~30%
Fign-sgRNA2		150618–150637	TTGCACAGCGCTGGCCTCCT		~30%
Fign-sgRNA3		150660–150679	CCAACCCTGGTGCCCAGCTA		~30%
Fign-sgRNA4		150719–150738	TCCAGCAGGGTATCCCCCAC		~30%
Fign-sgRNA5		150801–150820	TCAGGCATTGCTGCCCCCAC	785–804^*^	~30%
Fignl1-sgRNA1	*fignl1* [ENSDARG00000016427]	9947–9966	GGCCTTAGAGGTCCACCTAA	1245–1264^**^	~20%
Fignl2-sgRNA1	*fignl2* [ENSDARG00000057062]	99460–99479	GGCTATCAGAACAGCAGTGT	801–820^***^	~40%

# *Mutation measured by estimation based on sequencing of amplicon containing the targeted fragment*.

* *Calculated according to fign mRNA NM_001020575*.

** *Calculated according to fignl1 mRNA NM_001128751*.

*** *Calculated according to fignl2 mRNA NM_001214908*.

**Figure 1:**
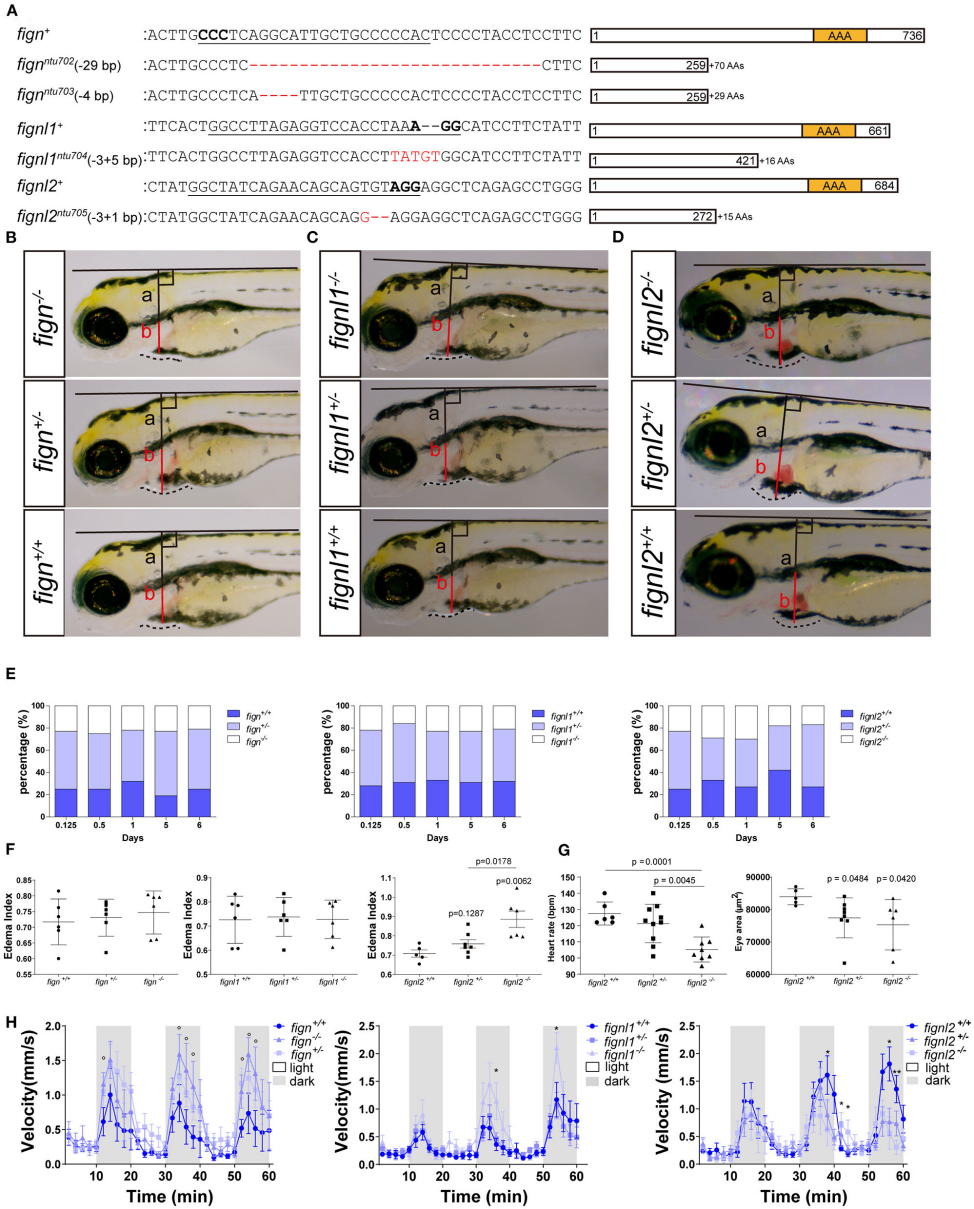
Loss of *fignl2* leads to smaller eyes, pericardial edema, and reduced swimming velocity. **(A)** Mutated alleles generated using CRISPR/Cas9 lead to complete loss of the AAA-type ATPase domain. Underlined sequences are the CRISPR targets, wherein the bases in bold are the proto-spacer adjacent motifs (sequences are shown only on the sense strand). **(B–D)** Representative phenotype observation and edema index measurement in Fign mutants **(B)**, Fignl1 mutants **(C)**, and Fignl2 mutants **(D)** at 4 dpf. **(E)** Percentage of the filial embryos generated by crossing of *fign*^+/*ntu*702^ × *fignl2*^+/*ntu*702^, *fignl1*^+/*ntu*704^ × *fignl2*^+/*ntu*704^ and *fignl2*^+/*ntu*705^ × *fignl2*^+/*ntu*705^, showing partial embryonic lethality of *fignl2* loss-of-function. Significance of differences compared to the wild type group is shown as p values on top of each dataset, and those between different mutant genotypes are shown on a horizontal line indicating groups used for comparison. **(F)** Statistics of pericardial edema index (PEI), *n* = (6,6,6) and (6,6,6) and (5,8,6); *p < 0.05, showing that the severity of edema was negatively correlated with functional *fignl2* alleles. **(G)** Heart rate of the filial embryos generated by crossing of *fignl2*^+/*ntu*705^ × *fignl2*^+/*ntu*705^ showing abnormalities in the cardiovascular system in the *fignl2* mutants, *n* = 6,10,8. Eyes of the filial embryos generated by crossing of *fignl2*^+/*ntu*705^ × *fignl2*^+/*ntu*705^ showing abnormalities in the cardiovascular system in the *fignl2* mutants, *n* = 5,8,6. **(H)** Statistics of swimming velocity with 10-min light-dark cycle of *fign, fignl1*, and *fignl2* mutants at 5 dpf, *n* = (12,25,9) and (14,22,11), and (20,19,9), showing weakened swimming ability of *fignl2* mutants. Circles indicate a *p* < 0.1, asterisks (*) indicate a *p* < 0.05, and (**) indicates a *p* < 0.01, as indicated by the Student's *t*-test between the wild type and homozygous mutant groups.

*fign* sgRNA1-5 were able to induce ~30% indel mutations in injected zebrafish embryos at 24 hpf. sgRNA5 was used to generate mutants for the following studies, producing mutated alleles *ntu702* and *ntu703* (Figure 1A), resulting in a protein completely missing the AAA domain (Figure 1A). *fignl1* sgRNA1 induced a mutation level of 20% in 24 hpf embryos, generating the mutated allele *ntu704* (Figure 1A), leading to a complete loss of the AAA-type ATPase domain. *fignl2* sgRNA1 was found to be active with a somatic mutation rate of 30–40% in the founders at 24 hpf. One mutated allele was found to have a deletion of 3 bp and an insertion of 1 bp and was named *ntu705* (Figure 1A), resulting in complete loss of AAA domain (Figure 1A).

F2 embryos were imaged for morphological changes at 4 dpf and behavioral analysis at 5 dpf and collected for genotyping to identify any phenotype caused by loss-of-function of *fignl2*. Embryos with *fignl2* mutations were found to have pericardial edema and smaller eyes, and the phenotypes were dependent on gene dose because homozygous mutants showed more severe edema and heart congestion (Figure 1D), while Fign and Fignl1 mutants showed no significant phenotypes in the pericardial area and the heart (Figures 1B,C). Cardiovascular problems may lead to embryonic death; thus, we checked the survival of embryos with mutated *fignl2*. The ratios of genotypes at 5 and 6 dpf were far from the Mendelian segregation, where the percentage of homozygous mutants was significantly reduced, demonstrating that the survival ability of mutants was weakened by the mutation and suggesting developmental abnormalities caused by loss-of-function of *fignl2* (Figure 1E). However, no similar reduction in the number of homozygous mutants during development was observed in *fign* or *fignl1* mutants (Figure 1E). Homozygous mutants had greater pericardial edema index (PEI) compared to wild type and heterozygous mutant siblings (Figure 1F). *fignl2* depletion leads to phenotypes with a lower heart rate and smaller eyes (Figure 1G), in addition to pericardial edema. Then, the mutants and wild type siblings were imaged for behavioral analysis using light stimuli. Larvae were treated with a 10-min light and 10-min darkness cycle after an initial 30-min adaptation in darkness. The *fignl2* homozygous mutants responded to the light-dark shift with a lower swimming velocity than wild type siblings (Figure 1H), whereas *fign* and *fignl1* knockout mutants were able to swim faster than wild type siblings.

### *fignl2* Is Highly Expressed in Vascular Endothelial Cells and Neuronal Cells in Zebrafish Compared With *fign and fignl1*

**Figure 2:**
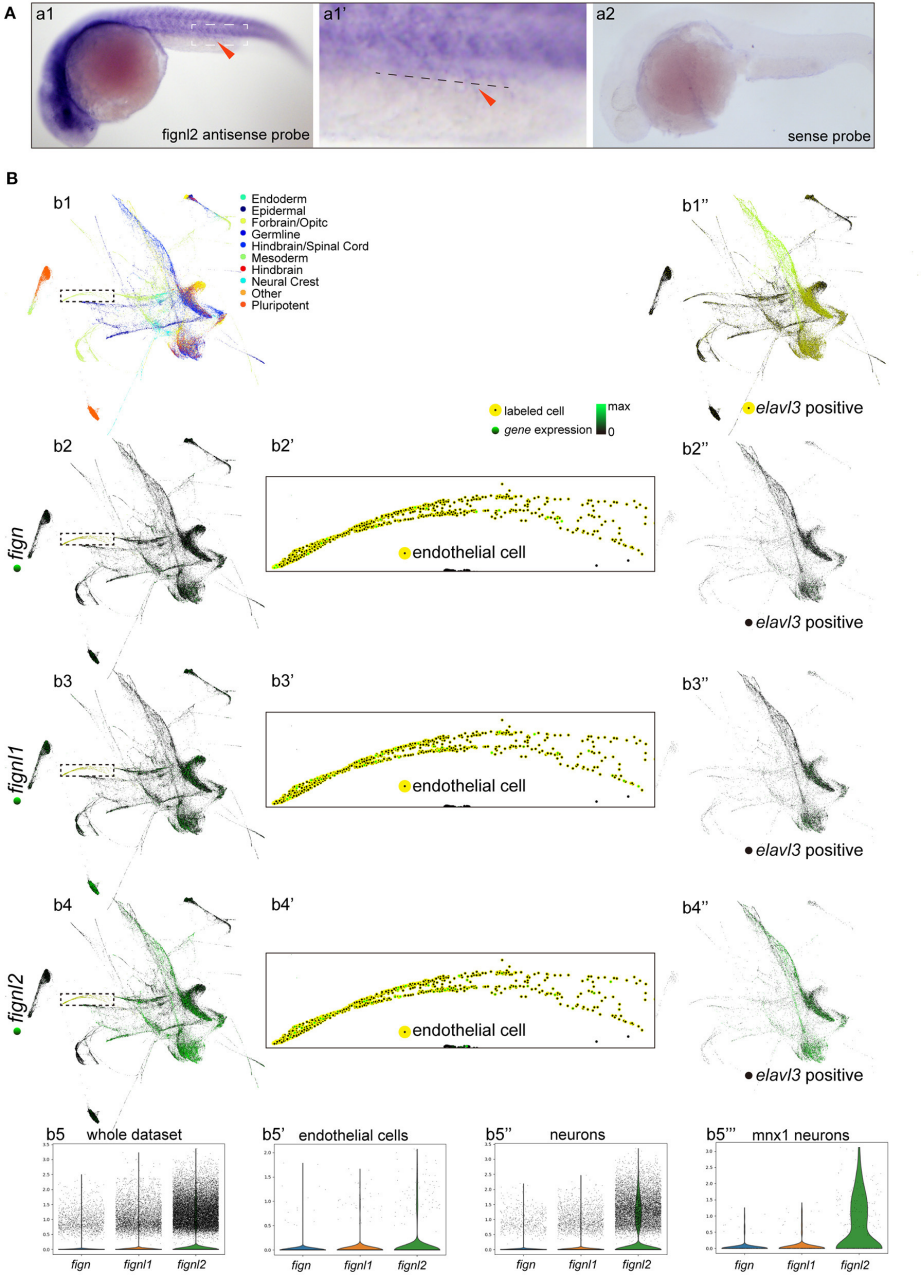
Expression of *fign, fignl1*, and *fignl2* in zebrafish embryos. **(A)** Expression analysis of *fignl2* using *in situ* hybridization. (a1) *fignl2* expression is observed in the nervous system and somites at 24 hpf, and weak staining is also observed in caudal blood vessels (arrowhead) (Enlarged in **a1′**). **(a2)** *in situ* hybridization using a *fignl2* sense probe as control. **(B)** Single-cell expression analysis of *fign, fignl1*, and *fignl2* during zebrafish development (a re-analysis of GEO GSE112294). **(b1)** Single-cells in the dataset (4–24 hpf) are mapped to different tissues. **(b2–b4)** Expression of *fign, fignl1*, and *fignl2* in the single-cell dataset. **(b5)** Violin plot of *fign, fignl1*, and *fignl2* expression in the single-cell expression dataset of zebrafish embryos (4–24 hpf). **(b2′-b4′)** Expression of *fign, fignl1*, and *fignl2* in endothelial cells (18 and 24 hpf). **(b5′)** Violin plot of *fign, fignl1*, and *fignl2* expression in endothelial cells (18 and 24 hpf). **(b1′′)** Neuronal cells were isolated by *elavl3* expression. **(b2′′-b4′′)** Expression of *fign, fignl1*, and *fignl2* in *elavl3*-positive cells. **(b5′′)** Violin plot of *fign, fignl1*, and *fignl2* expression in *elavl3*-positive cells. **(b5′′′)** Violin plot of *fign, fignl1*, and *fignl2* expression in *mnx1* positive cells. Expression data for violin plots are normalized and presented as counts per 10^4^.

As the mutants had problems in the cardiovascular system, and the Fign homologs were not previously described to be expressed in the heart and vessels, we then examined the expression of *fignl2* during zebrafish development using *in situ* hybridization. At 24 hpf, *fignl2* was mainly observed in the midbrain-hindbrain boundary, hindbrain, and somites as well as in the eyes and pectoral fins. Interestingly, in the family of Fign homologs in zebrafish, only *fignl2* was detected at a low level in the caudal vessels using *in situ* hybridization (Figure 2A), whereas *fign* and *fignl1* were not detected.

Due to the limitation of sensitivity of *in situ* hybridization, we utilized the online resource of single-cell sequencing data of zebrafish embryos (Gene Expression Omnibus accession number GSE112294) (Wagner et al., 2018) to check the expression of *fignl2*. According to the original research, the single cells were mapped to the forebrain, midbrain, hindbrain, and other tissues. This dataset provided an abundance of resources containing the most systematic temporal expression information, and more importantly, the most in-depth sequencing, allowing analysis of genes expressed at low levels.

We then focused on verifying the expression of *fignl2*, especially in endothelial cells. We analyzed *fignl2* expression during zebrafish development (along with its two paralogues, Figures 2Bb2–5) and labeled cells expressing *fignl2* from the dataset (Figure 2Bb4). Cells expressing *fignl2* were found in all brain regions and somites. Interestingly, when we highlighted endothelial cells at 18 and 24 hpf; many of these cells showed *fignl2* expression (Figure 2Bb4′). According to the clustering results in the original research, cells expressing *fignl2* include neural stem cells, neurons, epithelial cells, somite cells, and endothelial cells. Further analysis of the transcript levels of *fign, fignl1*, and *fignl2* in the cluster of endothelial cells revealed that the transcript amount of *fignl2* is remarkably more than those of *fign* and *fignl1* (Figures 2Bb2′–b5′).

Using the same method, we distinguished the cluster of neuronal cells according to *elavl3* expression and displayed the neuronal cells isolated from the dataset (Figure 2Bb1′′). The *fign, fignl1*, and *fignl2* transcripts are shown in Figures 2Bb2′′–b4′′ and a violin plot (Figure 2Bb5′′), and the results clearly showed that *fignl2* had significantly higher transcript levels in neuronal cells than did Fign or Fignl1. Given the *fignl2* mutants with a lower swimming velocity, we also extracted the expression data of *mnx1*-positive motor neurons and analyzed the transcript levels of *fign, fignl1*, and *fignl2* (Figure 2Bb5′′′), and the results showed that the level of *fignl2* transcripts was notably higher than those of *fign* and *fignl1*.

The above results showed that *fignl2* is expressed in more tissues with high levels than its paralogous genes, which may explain why *fignl2* null mutant zebrafish displayed serious abnormal phenotypes.

### Loss of *fignl2* Caused Abnormalities in Branching in Endothelial Cells and Neurons

**Figure 3:**
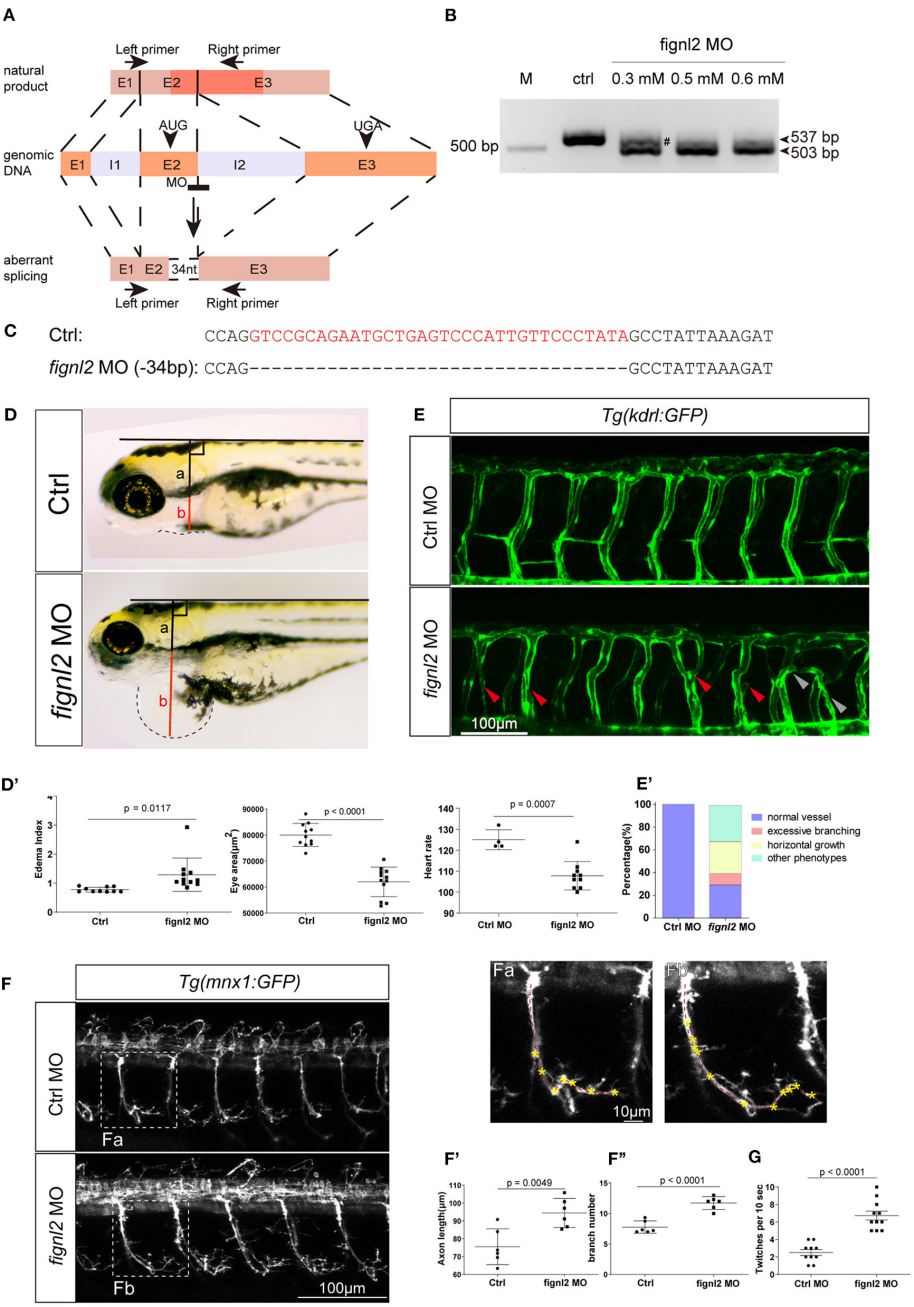
Depletion of *fignl2* causes morphological changes in intersegmental vessels (ISVs) and caudal primary neurons. **(A)** Schematic diagram of morpholino oligonucleotide (MO) induced *fignl2* knockdown causing aberrant splicing. **(B)** MO treatment results in mRNA size change in *fignl2* RT-PCR amplicon. The pound sign (#) shows some amplicons of the original size detected after treatment with 0.3 mM MO. **(C)** MO treatment results in a 34-bp deletion in *fignl2* mRNA. **(D)** *fignl2* knockdown causes pericardial edema in developing zebrafish. Graphical representation of edema index **(D′)**, eye size **(D′′)**, heart rate **(D′′′)** in Ctrl and *fignl2* knockdown zebrafish. Ctrl MO, *n* = 10; *fignl2* MO, *n* = 11. **(E)** *fignl2* knockdown causes morphological changes in ISVs, arrows showing malformations. Graphical representation **(E′)** of the percentage of different form of ISV malformations caused by *fignl2* knockdown. Ctrl MO, *n* = 10; *fignl2* MO, *n* = 11. **(F)** *fignl2* knockdown causes morphological changes in caudal primary neurons, including greater axon length and increased branching. Representative axons were shown enlarged in (Fa) and (Fb). Graphical representation of axon length **(F′)**, primary branch numbers **(F′′)** in Ctrl and *fignl2* knockdown zebrafish. Ctrl MO, *n* = 10; *fignl2* MO, *n* = 11. **(G)** *fignl2* mutants shows more frequent twitching. Twitching was counted in 10 s. Ctrl MO, *n* = 10; *fignl2* MO, *n* = 11.

We then wanted to confirm whether the loss of Fignl2 indeed resulted in heart defects in zebrafish. Genomic gene defects may cause some secondary effects to alter developmental features; thus, we performed gene knockdown using morpholino oligonucleotides (MOs) to determine if similar phenotypes, that is, a phenocopy of the mutants, could be generated. Splicing-blocking MO was designed to target the splicing acceptor of intron 2 in *fignl2* (Figure 3A), and a series of doses of the MO were tested. The *fignl2*-MO resulted in partial (0.3 mM) or complete (0.5 and 0.6 mM) deletion of a 34 bp fragment in the coding sequence due to activation of a cryptic splice site (Figures 3B,C). Then, 0.5 mM splice-blocking MO was injected into 1-cell stage Tübingen (Tu) zebrafish embryos. The phenotypes observed in the *fignl2* mutants also appeared in these morphants. At 4 dpf, *fignl2* morphants displayed pericardial edema and reduced eye size. *fignl2* morphants with pericardial edema had a lower heart rate than did those injected with control MO (Figures 3D–D′′′). Morphants with higher levels of MO displayed more severe phenotypes (data not shown).

Pericardial edema suggested abnormalities in the cardiovascular system during development. To further understand the function of *fignl2* in zebrafish development, we generated *fignl2* morphants using MO in transgenic zebrafish *Tg(kdrl:GFP)* labeling endothelial cells (Jin et al., 2005). *fignl2* MO was injected into 1-cell stage *Tg(kdrl:GFP)* embryos, and the embryos were used for morphological observation and blood vessel imaging at 48 hpf.

Intersegmental blood vessels (ISVs) in *fignl2* morphants showed more branching and an improper direction of growth. Some ISVs were linked to a neighboring ISV to form a Y shape or a circle (Figures 3E,E′), which was observed in both segmental arteries and veins, identified by whether they connected to the dorsal aorta or posterior cardinal vein (Ellertsdottir et al., 2010). Fignl2 is known to be a member of the AAA-type ATPase family with microtubule-severing function, and it is involved in regulating microtubule behaviors such as cellular pseudopodia extension and branching. We injected CRISPR/Cas9 in different combinations to determine whether the *fign* family members differ in their function in regulating endothelial branching. Co-injection of Cas9 mRNA and sgRNAs targeting *fign* and *fignl1* caused minor defects in ISV branching, and in the embryos that received co-injection of CRISPR/Cas9 targeting *fign, fignl1*, and *fignl2*, the morphological changes seemed to be mainly contributed by mutation of *fignl2* (Supplementary Figure 1A).

Microtubule dynamics are essential not only in endothelial branching, but also in the development of other cell types, especially in axon extension, and branching in neurons. Since the loss-of-function of Fignl2 affects branching in endothelial cells, it may cause changes in neurons where it is not only expressed but at a higher level. We performed similar treatments and observations at 28 hpf in *Tg(mnx1:GFP)* (Flanagan-Steet et al., 2005) (caudal primary neurons expressing GFP) for the effect of *fignl2* knockdown, and the loss of *fignl2* led to elongated axons and increased the number of branches in *mnx1*-expressing neurons (Figures 3F–F′′′) at 28 hpf and at 5 dpf (Supplementary Figure 2). Co-injection of Cas9 mRNA and sgRNAs targeting *fign* and *fignl1* reduced neurite length, which was consistent with the reported observation with MO targeting *fignl1* (Fassier et al., 2018). However, loss of *fignl2* resulted in longer neurites and more branching, and a similar result was observed in the embryos that received injection of CRISPR/Cas9 targeting *fign, fignl1*, and *fignl2* (Supplementary Figure 2).

Twitching of zebrafish embryos represents a type of spontaneous movement (Muto et al., 2011). We observed and recorded, and unexpectedly found that the spontaneous twitching at 28 hpf was more frequent in *fignl2* morphants (Figure 3G and Supplementary Video 1).

## Discussion and Conclusion

As members of the AAA ATPase family*, fign* paralogous genes play various roles, and their functions are relatively poorly understood for a short research history. We previously investigated and compared the expression patterns of *fign, fignl1*, and *fignl2* during zebrafish development and found that Fignl2 is expressed at higher levels in the nervous system and other tissues than *fign* and *fignl1* genes, which is consistent with the result of re-analyzed online single-cell RNAseq data (GEO GSE112294) (Wagner et al., 2018). Furthermore, *fign, fignl1*, and *fignl2* were also shown to be expressed in zebrafish endothelial cells. However, knockout of *fign* or *fignl1* did not yield obvious phenotypes, whereas depletion of *fignl2* (at the genomic DNA level or mRNA level) caused serious zebrafish defects such as pericardial edema, reduced heart rate, and reduced swimming velocity. These hinted that Fignl2 may have some important functions, which Fign or Fignl1 fails to compensate, but not *vice versa*. We further used GFP-labeled vascular endothelial cells or motor neurons to reveal that *fignl2* knockdown resulted in abnormal vascular branching as well as longer axonal length and more branches of caudal primary neurons, clarifying that Fignl2 regulates cellular branching. The pericardial edema or the weakened reaction to the lighting shifts in Fignl2-depleted zebrafish may be due to abnormal branching in endothelial cells or neurons. Twitching behavior, the first behavior in developing zebrafish, was used to evaluate the activity of the motor circuit (Muto et al., 2011). Twitching was more frequent in the *fignl2* morphants (Figure 3G and Supplementary Video 1), suggesting that depletion of *fignl2* impaired spontaneous movement, while the decreased response after light-to-darkness shift suggested changes in vision-related circuits in the *fignl2* morphants.

Although there were some differences between the morphants and the mutants, which could have been caused by genetic compensations (Rossi et al., 2015), they exhibited similar changes after knock down or knockout. These results, together with those of the rescue experiment using *fignl2* mRNA, indicate that the phenotypes seen in the endothelial cells and *mnx1* neurons are *fignl2*-specific.

Cellular branch formation is a universal morphological change during development. In developmental processes, including angiogenesis and neurogenesis, microtubule dynamics regulates cell branching morphogenesis by mediating branch orientation or extension (Liu et al., 2010; Myers et al., 2011; Hu et al., 2012; Lyle et al., 2014; Dong et al., 2019). This study presented cellular branches alteration in morphology of ISVs and CaP neurons. Microtubule severing proteins like Fign homologs strongly affect microtubule length and direction in neurons (Leo et al., 2015; Tao et al., 2016; Fassier et al., 2018; Matamoros et al., 2019) and other cell types (Hu et al., 2017), and our study offered clear data that Fignl2 is involved in cell branching.

Our previous study showed that *fign, fignl1*, and *fignl2* are similar in their high expression in the central nervous system during zebrafish early development, while fignl2 is also expressed in other tissues, e.g., pronephros, where fign and fignl1 are not detectable (Dong et al., 2021). The difference in expression patterns indicate figns function during cell branching may have differentiated. Although animals with ablated *fign, fignl1*, and/or *fignl2* tend to swim fast under constant lighting, *fignl2* null mutants responded to “light to darkness shift” more weakly than their wildtype or heterozygous siblings, indicating this change in vision-related behavior resulted from eye defect due to *fignl2* loss of function.

In summary, this study provided comparative results of the preliminary functional analyses of *fign, fignl1*, and *fignl2* in one zebrafish developmental system. Here, we report the finding that the microtubule-severing protein, Fignl2, contributes to proper cell branching during endothelial and neuronal development.

## Materials and Methods

### Zebrafish Husbandry

Zebrafish were housed in the Zebrafish Center at Nantong University. Zebrafish embryos of Tübingen and *Tg(flk:GFP)* were obtained through natural mating and maintained at 28.5°C. Embryos older than 24 h post-fertilization (hpf) were treated with 0.2 mM 1-phenyl-2-thio-urea (PTU, Sigma P7629, a tyrosinase inhibitor commonly used to block pigmentation and aid visualization of zebrafish development). Breeding of *fign* mutant zebrafish was performed by Nanjing YSY Biotech Company Ltd.

### Bioinformatics

The zebrafish *fignl2* genomic information was obtained from GenBank (Gene ID: 561837, mRNA NM_001214908.1, protein NP_001201837.1). Conserved domains of the *fignl2* proteins were localized according to the Ensembl database (http://www.ensembl.org/). *fignl2* sequences were aligned using Vector NTI software (http://www.thermofisher.com/). The phylogenetic tree was generated using MEGA X (https://www.megasoftware.net/) from aligned sequences generated with Clustal W (https://www.genome.jp/tools-bin/clustalw). The zebrafish embryo single-cell sequencing dataset (Wagner et al., 2018) was obtained from (https://kleintools.hms.harvard.edu/paper_websites/wagner_zebrafish_timecourse2018/mainpage.html) and visualized using SPRING (Weinreb et al., 2018). The run.py script for data preparation was modified to import original clustering results for visualization and is provided as Supplementary Document 1. Expression analyses were performed using Scanpy 1.5.1 (https://github.com/theislab/scanpy/) in a Python 3.8.3 environment, where neurons were extracted using the marker gene *elavl3*, and *mnx1*-positive neurons were extracted regarding *mnx1* expression. Expression data of endothelial cells were extracted according to the original clustering results provided in the dataset. The Python script is provided in Supplementary Document 2.

### Morpholino and Microinjection

The sgRNA templates were prepared by PCR with a forward primer composed of the first 17 bases of minimum T7 promoter followed by 20 bases identical to target proto-spacer and 20 bases identical to the first 20 bases of sgRNA scaffold, a reverse primer complementary to the last 25 bases of sgRNA scaffold (Table 2) and a template plasmid pT7-gRNA kindly provided by Prof. Bo Zhang at Peking University. The PCR products were used as templates for *in vitro* transcription using MAXIscript T7 Kit (Invitrogen, USA) to obtain sgRNAs. Capped Cas9 mRNA was prepared by *in vitro* transcription using mMessage mMachine T7 Kit (Invitrogen, USA) with a zebrafish optimized Cas9 template plasmid pGH-T7-zCas9 (Liu et al., 2014) kindly provided by Prof. Bo Zhang at Peking University. Cas9 mRNA and sgRNA were mixed and adjusted to a final concentration of 300 ng/μl:100 ng/μl. The MOs were synthesized by Gene Tools Company. MO antisense oligomers were prepared at a stock concentration of 1 mM according to the manufacturer's protocol. The sequence of zebrafish *fignl2* splicing MO in this study was 5′-TCAGAAATGTAGCACTTACTATAGG-3′ and the standard control MO was 5′-CCTCTTACCTCAGTTACAATTTATA-3′. MOs or mixtures containing 300 ng/μl Cas9 mRNA and 100 ng/μL sgRNA were injected into *Tg(flk:EGFP)* or *Tg(mnx1:EGFP)* embryos at 1 nL of solution per embryo using borosilicate glass capillaries (Sutter, USA) pulled using a P-97 micropipette puller (Sutter, USA) and connected to an IM-400 pneumatic microinjector (Narishige, Japan).

**Table 2:** PCR primers for gRNA template preparation and genotyping of the mutants produced in this study.

**Primer**	**Sequence (5^**′**^-3^**′**^)**
T7-gRNA-primer-F	TAATACGACTCACTATA + proto spacer + GTTTTAGAGCTAGAAATAGC
gRNA-primer-R	AAAAAAAGCACCGACTCGGTGCCAC
fignl2-genotyping-F	GCAGCTCCTGCAGTGGGCCCCATTC
fignl2-genotyping-R	CTCTTCTAATGCTGCTTTAATGTG
fignl2-seq-F	TCAACCGAACCCTACACTTC
fign-genotyping-F	GCTGTGCTGCAGATGTGATT
fign-genotyping-R	GGCTTAAACGCCAAAGATGA
fign-seq-F	CACCTCCAGATGTGACAGCA
fignl1-genotyping-F	TTGAAGGACAACACACCAAAAGATA
fignl1-genotyping-R	TACAGTATAGGCCGAACC
fignl1-seq-R	TACAGTATAGGCCGAACC

### RNA Extraction, Reverse Transcription, and RT-PCR

Tissue was homogenized and frozen in TRIzol UP (TransGen Biotech, Beijing, China) and stored at −80°C. Total RNA was extracted following the manufacturer's instructions. RNA (1 μg) was reverse-transcribed into cDNA using the HiScript II 1st Strand cDNA Synthesis Kit (Vazyme, Nanjing, China) according to the manufacturer's instructions. Synthesized cDNA was stored at −20°C.

### Whole Mount *in situ* Hybridizations

The 24 hpf cDNA served as templates for cloning *fignl2* fragments to make antisense RNA probes for zebrafish *fignl2*. The *fignl2* primers for RT-PCR were: Left primer, 5′-TCCTGCTATTTGGCCCTCAA-3′; Right primer, 5′-ACAACTCCCTTTCGCTGAGA-3′; the amplicon is a 441 bp fragment in the coding sequence for *fignl2*. Digoxigenin (DIG)-labeled RNA sense and antisense probes were made from the linearized plasmids using the DIG RNA Labeling Kit (SP6/T7) (Roche). Whole mount *in situ* hybridization was performed following the methods as previously described (Schulte-Merker et al., 1992). The concentration of probes was 2 ng/μl in fresh hyb^+^, and for blocking purpose, 2% blocking reagent (Boehringer blocking reagent, Roche), 10% sheep serum (Sigma), and 70% MAB (YSY, Nanjing, China) were used.

### Imaging

For confocal imaging of blood vessel development in *Tg(flk:GFP)* zebrafish and the neurons in *Tg(mnx1:GFP)* zebrafish, embryos were anesthetized with egg water/0.16 mg/mL tricaine (Sigma E10521)/1% 1-phenyl-2-thiourea (Sigma P7629) and embedded in 0.6% agarose. Images were taken using a Leica TCS SP5 LSM confocal microscope (Leica, Wetzlar, Germany). The analysis was performed using Imaris (http://www.bitplane.com/). For behavioral studies, zebrafish juveniles were imaged using the Noldus DanioVision system and analyzed using EthoVision XT software (https://www.noldus.com/). Animals were placed in a 48-well plate, each juvenile occupying a well. Before the beginning of detection, the light was kept off for 30 min, followed by three tandem 10 min light/10 min darkness cycles with video recording for light/dark shift behavior studies, or 15 min with the light on for spontaneous behavior observation.

### Measurement and Statistics

Axon length and branching were measured and counted as previously described (Dong et al., 2019). We measured the extent of pericardial edema by defining a PEI: from the center of the pericardium (P), a perpendicular to the extended back midline was made and let the intersection be A; let the distance from P to A be a and the radius of the pericardium be b; the pericardial edema index is defined as b/(b-a). All data analysis, statistical comparisons, and graphs were generated using GraphPad Prism 5 (http://www.graphpad.com/scientific-software/prism/). Significance of differences was analyzed using Student's *t*-test. Data are expressed as mean ± S.E.M. (standard error of the mean).

## Data Availability Statement

The datasets presented in this study can be found in online repositories. The names of the repository/repositories and accession number(s) can be found in the article/Supplementary Material.

## Ethics Statement

The animal study was reviewed and approved by Ethics Committee on Animal Experimentation of Nantong University.

## Author Contributions

ZD, XC, YL, RZ, and XL carried out the experiments, data collection, and analysis. ZD and ML prepared the manuscript. ZD and ML contributed to study design and management. All authors contributed to the article and approved the submitted version.

## Conflict of Interest

The authors declare that the research was conducted in the absence of any commercial or financial relationships that could be construed as a potential conflict of interest.
