# Supplementary material for: Microtubule Severing Protein Fignl2 Contributes to Endothelial and Neuronal Branching in Zebrafish Development
Source: Front Cell Dev Biol. 2021 Jan 18;8:593234. doi: 10.3389/fcell.2020.593234 (PMC7873885; doi:10.3389/fcell.2020.593234)
Supplement: Supplementary file 5 [file Image_1.PDF]

Supplementary Data 1

Supplementary Figure 1
Supplementary Figure 2
Legend of Supplementary Video 1
Legend of Supplementary Video 2

Microtubule severing protein Fignl2 contributes to endothelial and neuronal branching in zebrafish development

Zhangji Dong[1], Xu Chen[1], Yuanyuan Li[1], Run Zhuo, Xiaona Lai, Mei Liu*

Key Laboratory of Neuroregeneration of Jiangsu and Ministry of Education, Co-innovation Center of Neuroregeneration, Nantong University; Nantong Jiangsu 226001, China

[1] These authors contributed equally to this work.

*Correspondence should be addressed to:

Mei Liu, Ph.D.

Professor,

Key Laboratory of Neuroregeneration of Jiangsu and Ministry of Education, Co-innovation Center of Neuroregeneration, Nantong University,

19 Qixiu Road, Nantong, Jiangsu 226001, China
E-mail: liumei@ntu.edu.cn

A

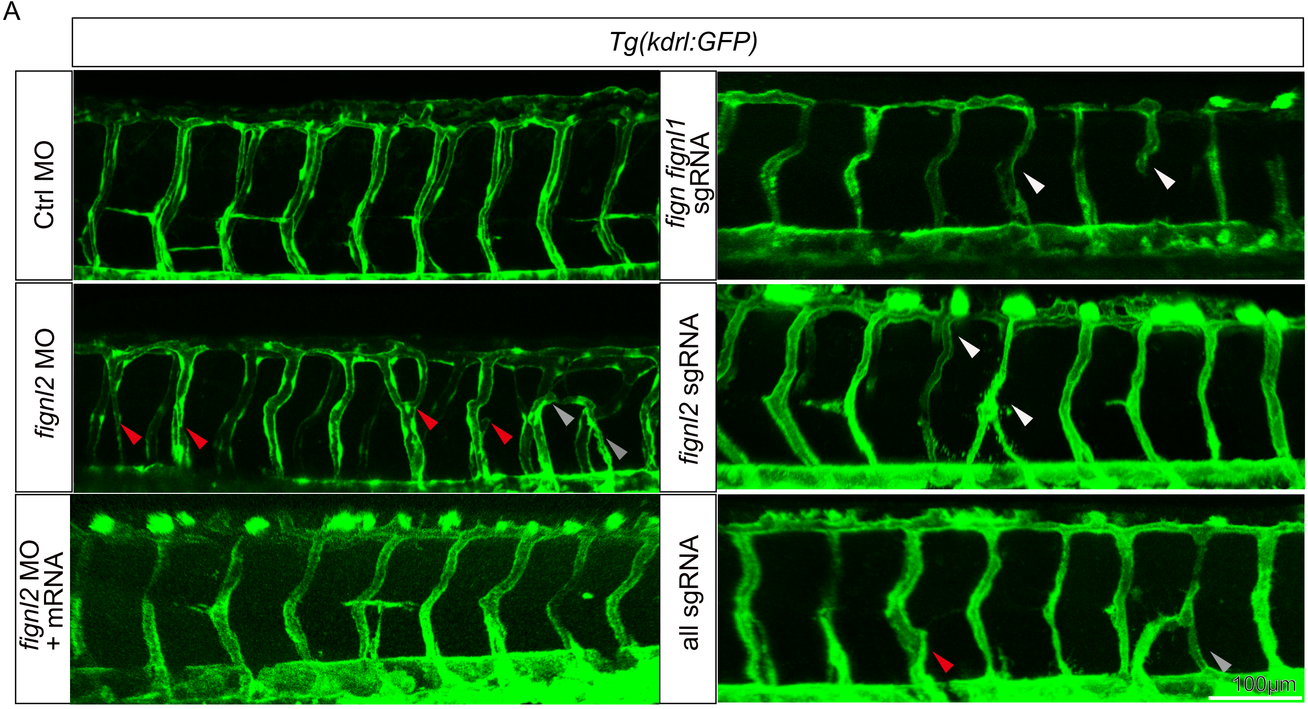
Tg(kdrl:GFP)
Ctrl MO
fign fignl1 sgRNA
fignl2 MO
fignl2 sgRNA
fignl2 MO + mRNA
all sgRNA
100µm

B

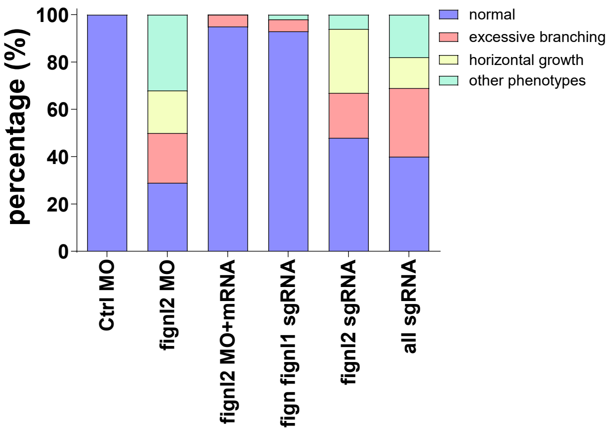
percentage (%)
100
80
60
40
20
0
normal
excessive branching
horizontal growth
other phenotypes
Ctrl MO
fignl2 MO
fignl2 MO+mRNA
fign fignl1 sgRNA
fignl2 sgRNA
all sgRNA

Figure S1. Abnormal branching in vascular endothelial cells after knockdown or mutation of *fignl2* in zebrafish.

(A) Defects in intersegment vessels (ISVs) after knockdown or mutation of *fignl2*. *fignl2* morphants display Y-shaped, short, or thin ISVs (arrowheads). Both segmental arteries (red arrowheads) and veins (gray arrowheads) are affected. *fignl2* mRNA can rescue the phenotype caused by *fignl2* MO. Mutations in *fign* and *fignl1* cause milder defects compared to those seen in *fignl2* mutants.

(B) Graphical presentation of phenotypes in morphants and mutants of *fign* family members in zebrafish.

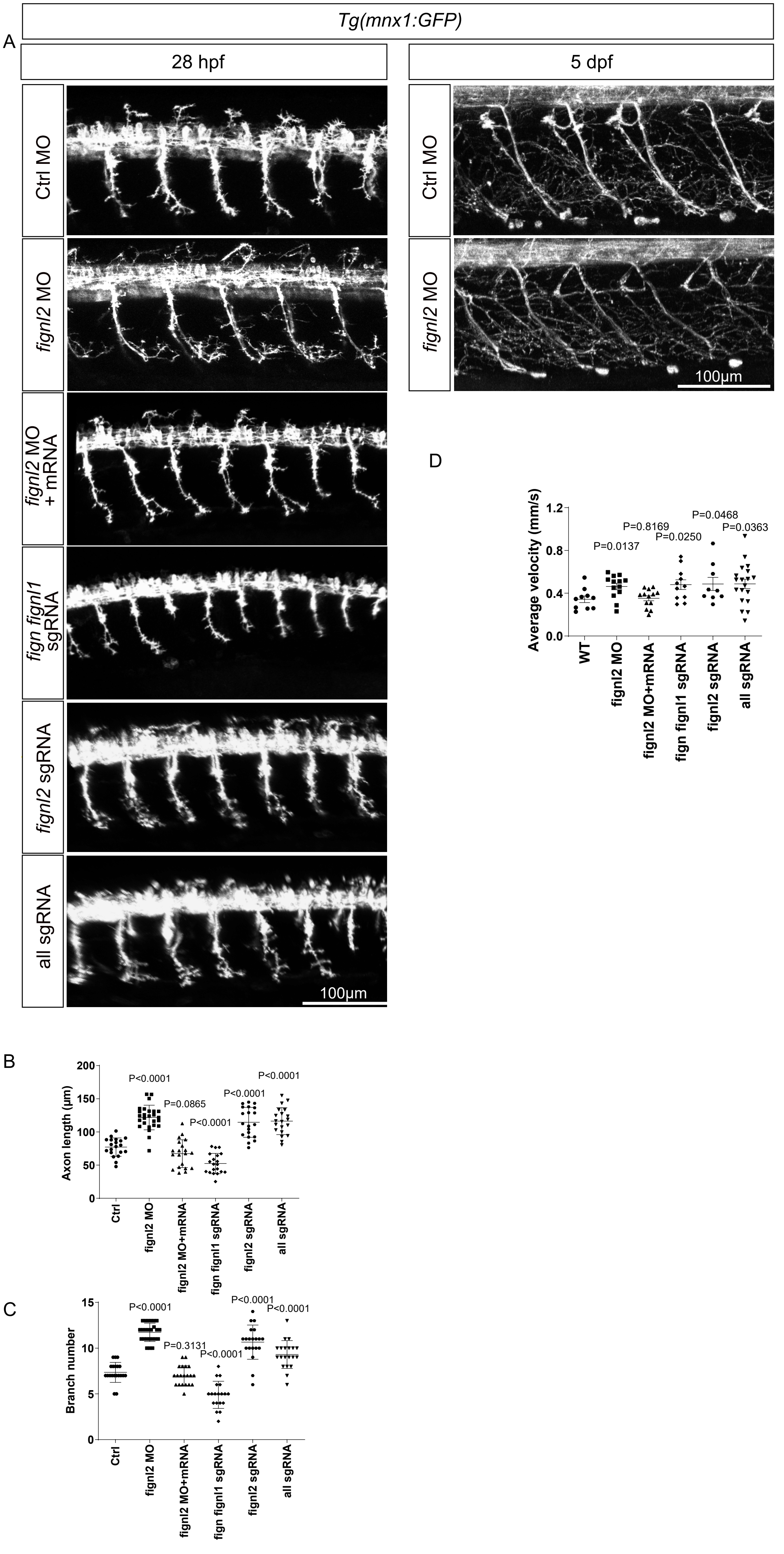
Tg(mnx1:GFP)
A
28 hpf
5 dpf
Ctrl MO
fignl2 MO
fignl2 MO + mRNA
fign fignl1 sgRNA
fignl2 sgRNA
all sgRNA
100µm
D
Average velocity (mm/s)
P=0.0137
P=0.8169
P=0.0250
P=0.0468
P=0.0363
WT
fignl2 MO
fignl2 MO+mRNA
fign fignl1 sgRNA
fignl2 sgRNA
all sgRNA
B
Axon length (µm)
P<0.0001
P=0.0865
P<0.0001
P<0.0001
P<0.0001
Ctrl
fignl2 MO
fignl2 MO+mRNA
fign fignl1 sgRNA
fignl2 sgRNA
all sgRNA
C
Branch number
P<0.0001
P=0.3131
P<0.0001
P<0.0001
P<0.0001
Ctrl
fignl2 MO
fignl2 MO+mRNA
fign fignl1 sgRNA
fignl2 sgRNA
all sgRNA

Figure S2. Abnormal branching caudal primary neurons and behavior changes after knockdown or mutation of *fignl2* in zebrafish.

(A) Knockdown or mutation of *fignl2* increases neurite length and branch numbers in caudal primary neurons. Mutation of *fign* and *fignl1* reduces axon length and branch numbers. Mutation of only *fignl2* or all of *fign*, *fignl1,* and *fignl2* shows similar effects to that seen in *fignl2* morphants at 28 hpf (left panel) and 5 dpf (right panel).

(B) Knockdown or transient mutation of *fign* family members in zebrafish alters the length of *mnx1* neurons at 28 hpf. Knockdown or mutation of *fignl2* significantly increases the length of axons, while mutation of *fign* and *fignl1* reduces the length.

(C) Knockdown or transient mutation of *fign* family members in zebrafish alters the branching of *mnx1* neurons at 28 hpf. Knockdown or mutation of *fignl2* significantly increases the branching of axons, while mutation of *fign* and *fignl1* reduces branches.

(D) Mutations in *fign* family members in zebrafish increases swimming velocity at 5 dpf.

Video S1. Zebrafish embryos were injected with control morpholino oligonucleotides or fignl2 morpholino oligonucleotides. *fignl2* morphants exhibited more frequent twitching at 28 hpf.

Video S2. Zebrafish morphants on *Tg(mnx1:EGFP)* background at 5 dpf showed longer axons and more branches in caudal primary neurons.
